# Nursing Leadership in a Post-Pandemic Elective Orthopaedic Theatre Department: A Detailed Thematic Analysis of an Open-Ended Qualitative Survey

**DOI:** 10.3390/nursrep14030116

**Published:** 2024-06-24

**Authors:** Carlo Biz, Lisa Buffon, Davide Scapinello, Sean Semple, Elisa Belluzzi, Ron Batash, Pietro Ruggieri

**Affiliations:** 1 Orthopaedics and Orthopaedic Oncology, Department of Surgery, Oncology and Gastroenterology DiSCOG, University of Padova, Via Giustiniani 3, 35128 Padua, Italy; davide.scapinello@studenti.unipd.it (D.S.); ron.batash@gmail.com (R.B.); pietro.ruggieri@unipd.it (P.R.); 2 Centre for Mechanics of Biological Materials, University of Padova, 35131 Padua, Italy; 3 King’s College Hospital, Orpington Elective Orthopaedic Theatres, Denmark Hill, London SE5 9RS, UK; l.buffon@nhs.net; 4 Guy’s Hospital, Main Theatres, Great Maze Pond, London SE1 9RT, UK; sean.semple@gstt.nhs.uk; 5 Musculoskeletal Pathology and Oncology Laboratory, Department of Surgery, Oncology and Gastroenterology DiSCOG, University of Padova, Via Giustiniani 3, 35128 Padua, Italy

**Keywords:** COVID-19, nursing, nursing leadership, orthopaedic surgery, post-pandemic, qualitative survey, thematic analysis

## Abstract

Background: The COVID-19 pandemic has impacted nursing theatre staff, departmental activity, and delivery of services to patients. This work-based project aimed to investigate the challenges of nursing leadership in an elective orthopaedic department at current times. Methods: The study collected qualitative data exploring theatre staff’s expectations from leadership, offering insight on how the pandemic has influenced the way of working and exploring how the future in this unit may look. The answers from 20 practitioners to an anonymised open-ended survey were examined using thematic analysis. Results: The participants described a leader as a good communicator who focuses on empowering others and supporting the team, identified by the majority as a senior team member. From the findings, three topics were identified: immediate changes, delayed changes, and pre-existing conditions. The answers painted a reality that is complex and multifaceted, where numerous variables play a part in the physical and mental health of each candidate, impacting their performance as well as their work/life balance. Overall, the strongest subjects recurring in the findings were the need for nursing leadership to focus on supporting staff with training opportunities, to actively plan for a reduction in staffing shortages, and to be constantly mindful of staff well-being. Conclusions: This study pointed out that the need for constant communication with their staff, building honest relationships, and being a reliable leader, focused on empowering others and supporting the team were important factors for the nursing management during the COVID-19 pandemic and post-COVID-19 era.

## 1. Introduction

The Coronavirus disease (COVID-19) is an infectious disease caused by the severe acute respiratory syndrome coronavirus 2 (SARS-CoV-2), which spread worldwide, affecting more than 200 countries and with more than 770,000,000 cases reported since early 2020 by the World Health Organization [1]. Initially declared by the WHO a “Public Health Emergency of International Concern” (PHEIC) at the end of January 2020, and, subsequently, a pandemic, it has significantly overwhelmed healthcare infrastructure worldwide.

The impact of the global pandemic on theatre activities meant that all planned procedures had to be cancelled and postponed [2] to allow sick patients to access theatre facilities and equipment, prioritise the use of hospital beds, and redeploy staff to critical units [3]. In the UK, as a result, the National Health Service (NHS) in April 2020 announced that all non-urgent surgeries were suspended for at least three months.

According to the British Medical Association (2022), the health services across the UK entered the pandemic with a significant backlog of care, elongating the waiting period for diagnostics and elective care, while access to emergency care was deteriorating due to degeneration of symptoms previously addressed with elective procedures, with reduction in capacity and increased workload of up to 80% of the services [4].

The temporary suspension of surgery has resulted in three major issues, which directly impacted elective orthopaedic theatre departments:

(1) Delays in treatment caused increased patient waiting lists and patients’ care to become more complex for deteriorating diseases, such as arthritis (NHS England, 2022). The national data shows that 3.87 million patients had their operation within 18 weeks from referral, just 64% against the performance standard of 92% [5]. In addition to these figures, the House of Commons Committee (2022) estimates between 7.6 million and 9.1 million patients could be missing referrals for elective care [5];
(2) The need for services to change and adapt to the new reality. This includes the introduction of additional tests for patients, the need for services to be more flexible (e.g., clinics to quickly adapt to the changes and offer alternative options to face-to-face appointments), and the financial burden this process has on each healthcare setting [6];
(3) Staffing shortage, which was at critical levels before the pandemic (The King’s Fund, 2017) and it has worsened in the last years, due to the pandemic and redeployment or surge [3].

When healthcare professionals returned to their area after surging, they had to deal with the aftermath of a traumatic event impacting their well-being, the pressure of services to increase productivity and efficiency due to increased demand, while keeping up with constant changes in policies, practice, and challenging management of the most complex orthopaedic cases: the polytraumatic patients [7,8].

In the context of this study, the term “nursing leadership” refers to all managers (matrons and above) who can inspire, motivate, and lead staff towards agreed common goals in order to have a positive impact on staffing satisfaction and ultimately on patient outcome [9]. Over the years, numerous theories have been developed to understand the various dynamics of leadership and in the literature, there are different types of leadership such as shared, servant, ethical, hierarchical, and authentical leaderships [10,11,12,13]. Contemporary leadership theories emphasise collaborative, ethical, and adaptive approaches, reflecting the complexities and dynamic nature of the modern organizations, including the National Health Services.

This study aimed to explore and define the present challenges of nursing management in leading the theatre staff in an orthopaedic department, with four elective orthopaedic theatres after the COVID-19 pandemic.

We hypothesised that the findings of this study will impact the nursing leadership role, producing changes to the current services and addressing nursing issues. These will be to allow the nursing leadership team to offer better support to the theatre staff and indirectly improve the overall patient experience.

## 2. Materials and Methods

### 2.1. Study Design

The study employed an anonymised open-ended qualitative online survey (see Table 1), as the authors wanted to explore the staff’s feelings and ideas about specific issues and possible solutions. Three areas were assessed: exploring leadership, staff’s current work experience, and what the future should look like for nursing leadership.

The study was approved by the London South Bank University Ethical Committee, Ref. 22A09 on 23 May 2022 Approval was also given by King’s College Hospital by both the Head of Nursing on behalf of the Research and Development department on 27 January 2022 and the Lead Nurse for Theatres on 15 March 2022.

### 2.2. Setting, Recruitment, Participants, and Data Collection

The orthopaedic department is composed of four elective orthopaedic theatres with a staff of 36 registered nurses/operating department practitioners and healthcare assistants employed, with a turnaround of approximately 4500 patients being treated yearly.

The sample consisted of theatre staff, meaning the totality of the employees in the Theatre Department under nursing management (nurses, operating department assistants, and healthcare assistants). The sampling was non-probability, both convenient and purposive; it was easily accessible to the data collector [14] as the candidates were recruited from the data collector’s working environment, but it was also an effective and cost-effective method of recruitment [15], as they were highly qualified to answer the study question.

The recruitment was undertaken in a department with a total of 36 employees under nursing management, of which 26 were registered practitioners and 8 were unregistered staff.

The only inclusion criterion was the need for the staff to have been working in the department for at least 3 months at the moment of data collection, due to the limited exposure to the environment and the nursing leadership within the department (not in-depth knowledge).

The open-end survey was created on Google Forms. The employees were contacted via email, with useful information about the study and were asked to participate by replying with a signed consent form. Access to the survey was gained via a direct link sent via email to each participant. The survey was accessed, completed, and submitted in one go or in multiple phases, as progress could be saved.

The recruitment, consent, and data collection ran for approximately 6 weeks.

### 2.3. Data Analysis

The data were analysed using thematic analysis (TA). The software used was Taguette (https://www.taguette.org/ accessed on 1 January 2024) [16]. This study followed the reflexive TA, supported by Big Q values for the application of the technique in the qualitative paradigm, to identify and analyse themes, revealing the analyser’s subjectivity [17].

The authors grouped the answers to generate a coding table. Specifically, we considered the impact of the different aspects explored concerning the proposed question and based on the data analyser’s subjective knowledge and personal interpretation of the data collected.

The authors reflected on the data to reveal underlying ideas, assumptions, and conceptualizations, and to identify how the ‘fully realised’ shared themes flowed among the recurring concepts presented by the data collected.

Finally, the themes were compared with the objectives to answer the question of the study.

## 3. Results

Eight participants were excluded and out of the possible 28 eligible candidates, 20 (71.4%) voluntarily signed the consent form and returned a completed survey (20 out of 20). Among these, four (20%) were unregistered (two band 2 and two band 3) and 16 (80%) were registered practitioners (nine band 5, four band 6 and three band 7). The selected genders of the participants were seven males, twelve females, and one identified as “prefer not to say.”

All the answers to the survey received were accepted (none were discarded), even if two candidates omitted one question number.

The survey started by looking for a definition of leadership in the Orthopaedic Theatre Unit.

The findings showed that a third of the participants thought that leadership in the Orthopaedic Department was represented by a band 6 or above, while the others thought it was a mixture of different senior roles. A third of the candidates answered that they believed that leadership was not associated with a specific person or role.

The second question had similar results, with seven participants reported as band 6 and above as driving changes in the department and a quarter believed all staff were involved. Only three participants indicated that management leading changes could come from outside the theatre department.

**Figure 1:**
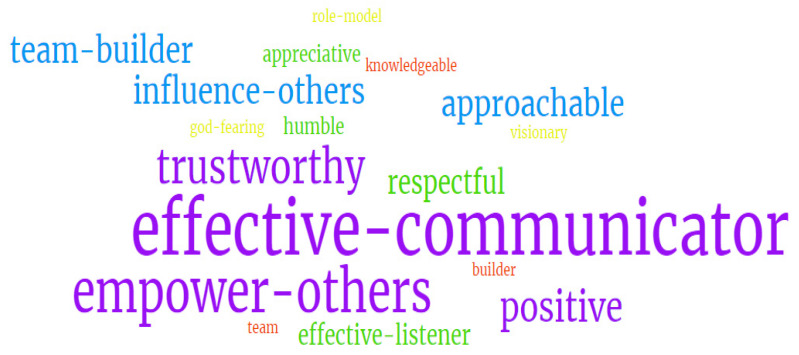
Word cloud of Question 3.

When the staff were asked to select the top three characteristics of a leader (Question 3), these were identified as: effective communicator (60%), empowering others (45%), and reliable/trustworthy (35%) (Figure 1).

**Figure 2:**
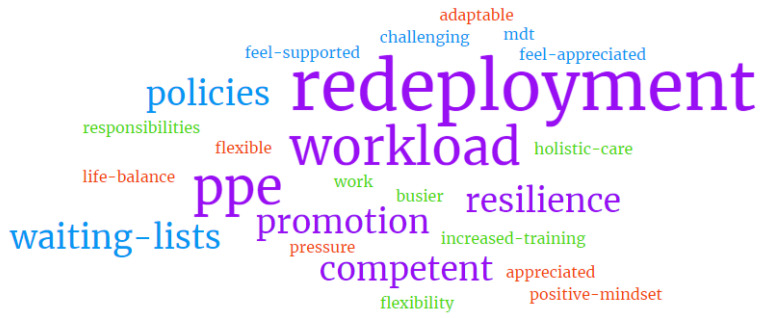
Word cloud of Question 4.

Redeployment was the main change impacting all participants following the pandemic (Question 4), followed by the increased workload and stricter use of PPEs (Figure 2). Staff also felt more opportunities for promotion were available, they gained new knowledge and were more competent, and they built resilience as a result of the challenges they had faced.

All the participants answered Question 5 affirmatively, mainly because of the increased workload, resulting in extended working hours, list overrunning, and feeling pressure to cover extra shifts.

Question 6 explored nursing management support, with the majority of the staff feeling listened to, encouraged, and empowered, even if few results highlighted feeling pressured at times and that management had aspirational views on unrealistic targets.

In order to improve the working environment, the majority of the participants suggested the need to focus on recruitment of skilled staff, receive more personalised feedback, respect others, and for mangers to be more involved as role models.

Answers to Question 8 were very varied, having promotion and pay rises as a main reason, followed by relocation, progression in other fields, and change of career (mainly as a result of mental health issues).

**Figure 3:**
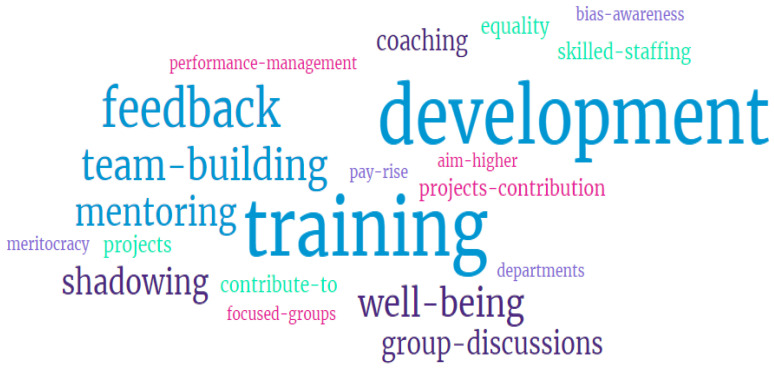
Word cloud of Question 9.

When asked how staff would improve their satisfaction and improve retention (Question 9), the majority (75%) said that “Training and Development” was the key for both (Figure 3).

After familiarisation, the topics were then reviewed and initial temporary themes were assigned, followed by classification into new groups, distinguishing themes, and sub-themes of the TA.

The thematic framework presents three distinct themes, which were each divided into four sub-themes each (Table 2), addressing theatre staff’s points of view on nursing leadership challenges in an orthopaedic department after the COVID-19 pandemic.

The first theme was named immediate changes. This included actions taken by leadership and consequences which affected the theatre staff as a direct result of the pandemic; these changes were quick and for the majority, mandatory.

The second theme was identified as all the delayed changes and this incorporated all actions which were caused by the pandemic, but only manifested when the activity (the elective procedures) restarted.

The third theme extrapolated was labelled pre-existing conditions and this grouped all the problems, which were already presented prior to COVID-19 but had worsened as a direct consequence of the suspension of all elective activity.

### 3.1. Immediate Changes

#### 3.1.1. Guidelines and Policies

Fourteen candidates reported the impact of constant changes in guidelines and policies on their daily work since the beginning of the pandemic, as local and national guidelines were constantly changing. Almost all the participants agreed that the pandemic had impacted practice for clinical and behavioural indicators, which sometimes influenced their mandatory requirement and their compliance levels.

#### 3.1.2. Personal Protective Equipment (PPE)

Eight accounts mentioned the introduction of PPE as part of the mandatory requirements of coronavirus management, including challenges connected to providing and distributing PPE across all the areas, the fit testing, and training of all staff not usually working in infected areas. The disproportion between supply and demand and the quick propagation of the pandemic also meant some staff had to care for patients infected with COVID-19 without the appropriate equipment (lack of available equipment) and the appropriate information (have attended fit testing sessions or having been trained on PPE wearing), putting themselves (and their family) at risk.

#### 3.1.3. Redeployment

All staff working in the elective theatres were moved to different departments wherever needed, such as critical areas, surgical wards transformed into intensive care, and high dependency and respiratory units. Some participants were moved to the emergency theatres or other so-called “green pathway wards” (wards free from COVID-19 patients), others were either shielded for medical reasons or assigned to the setup and running of the airway team at first and the vaccination centre. Band 7 and above were redeployed to management roles, but this decision meant that staff felt seniors were not on the frontline.

#### 3.1.4. Wellbeing

Staff Wellbeing was on the NHS agenda since the early phases of the pandemic, as stress, overwork, and exhaustion led many professionals to burn out and, in many cases, leave due to the pressure from the management to do overtime. Thirteen reported being unable to keep up with the personal duties of their family and friends and were worried to be the carriers of the disease which could impact their family. Furthermore, four candidates did not feel they were supported as they would have wanted to receive more personalised help.

### 3.2. Delayed Changes

#### 3.2.1. Increased Workload

Eighteen participants referred directly or indirectly to workload. As the elective theatres were closed and all activity was suspended, the orthopaedic elective department found itself with an increased workload at their reopening. Due to the backlog of elective procedures, the conditions of patients who had been waiting for a long time had worsened, and more complex procedures were needed. In theatre terms, this meant longer procedures (more theatre time, fewer numbers of patients per list), more equipment needed, and more skilled staff required to be able to manage the difficult cases.

#### 3.2.2. Organisational Pressure

The need to treat more patients, increase the workload, and optimise theatre time was linked with the determination of patients’ suitability criteria and the need for categorisation of their priority, which impacted theatre list but also theatres utilisation. Nine participants disclosed “feeling overwhelmed” from the pressure coming from the management, as they were requested to increase the prepandemic caseload and to deal at the same time with changes.

#### 3.2.3. Transfer or Change of Unit

In the attempt to clear the backlogs of patients on the waiting list for orthopaedic surgery, the division decided to move 13 trained practitioners with previous theatre experience (in the UK or from abroad) into theatres from other areas (wards or services).

#### 3.2.4. Physical Health

Nine staff members reported the impact of their work on their physical health. In their reports they mentioned being often physically exhausted and more likely to go off sick which could further aggravate the workload on the rest of the staff and even lead to serious mistakes.

### 3.3. Pre-Existing Conditions

#### 3.3.1. Staff Shortage

To recruit theatre staff into vacant positions, not only employees were moved from other areas, but also staff with no previous theatre experience were offered theatre positions. This meant the department had better establishment numbers, but not necessarily skilled staff.

#### 3.3.2. Training

The single action that emerged strongly as it was mentioned at least once by 90% of the participants. Due to the suspension of activities, including training, during the pandemic, many practitioners stated that they found themselves behind when the sessions restarted, with longer waiting times to get to the training and more likelihood to have their study day cancelled on the day of the training due to being recalled to the units. Training and development were indicated by the majority as the way to improve satisfaction and retention, and were identified as the preferred way to feel valued. Some also reported currently being involved in further education and attending university courses.

#### 3.3.3. Development and Support

Sixteen candidates agreed with the need to feel supported and should be actively involved in daily tasks, on the clinical field and away from the desk, as one of the most appreciated ways for managers to understand the issues in the department and support the staff, but also being good communicators and be approachable.

#### 3.3.4. Opportunities for Promotion

Twelve participants reported increased career opportunities for progression as new roles were created to deal with the new demands, even if the small environment meant not everybody could have the opportunity to get promoted.

## 4. Discussion

This study aimed to investigate the present challenges of nursing management in leading the theatre staff of an orthopaedic department.

For this study, an open-ended survey was chosen. The options of semi-structured interviews and focus groups were excluded to avoid disparity and willingness to share as the interviewer was the manager [18] and the difficulty in matching out-of-hours staff availability. As employees at different levels of seniority would be in the same focus group, this could have impacted the confidence in sharing. Another risk identified by the applicability of focus groups was the staff availability, including different shift patterns (participants being asked to come in on their days off), personal commitments (impacting the possibility to organise them after work), and time required (all participants should be present at the given time for the whole length of the activity). Similarly, these issues could have impacted the availability to meet for semi-structured interviews, while participants could complete the survey at their own leisure and dedicate as much or little time as preferred.

The findings were grouped into three themes, each one divided into four sub-themes, and all together narrating a picture of nursing leadership in the elective orthopaedic theatres with suggested insights.

This study presents qualitative data exploring each individual’s personal experience, with the intent to unveil the ‘theoretical-practical’ view of every participant and evaluate leadership performance. Some quantitative data were extracted to highlight the incidence and strengths of the positions in the context presented. The everyday shared experience was used to understand and describe the subjective view, while suspending the surveyor’s preconceived assumptions about the question of the study [19].

This study followed the reflexive TA to identify and analyse themes, revealing the analyst’s subjectivity. This final characteristic was very important as it clarified TA was not a neutral method, but instead, it presumed its analysis was influenced by the subjectivity of the authors, and this implied the personal interpretation of data. Terry et al. (2017) argued that many colleagues had the problematic assumption that the analyser’s subjectivity was flawed, and that better analysis would be achieved with a reduction in the influence of the analyst’s subjectivity [20].

A potential issue identified with this method was a poorly conducted analysis [21] (Braun and Clarke, 2006), but Nowell et al. (2017) suggested a structure to increase the trustworthiness of this model, by using precise and consistent data analysis, exhaustive and systematic recording of information, and detailing the methods of analysis to justify credibility [22]. Quality can impact the themes when an author uses the ‘fast and loose’ approach, with the risk of choosing the selection of data which fits the argument and not providing the best explanation or answer to the study question [20]. Another common mistake identified by Braun and Clarke (2021) was that too often authors use ‘coding reliability measures’ as universal requirements of quality TA, or as an assumption of homogeneity [23]. Gibson and Brown (2009) asserted that TA was not a specific analytic approach, but it was instead a meta-analytic technique, as most qualitative applications call for themes [24].

Coding means interpreting large segments of text and transforming them into information that can be grouped into themes and therefore compared with others with the same or similar label [25]. The authors grouped the answers to generate a coding scheme. Specifically, we considered the impact of the different aspects explored concerning the proposed question and based on the author’s subjective knowledge and personal interpretation of the data collected. The structure was shared by Campbel et al. (2013) who identified the need for standardisation of each unit and creating a coding scheme to reduce errors [26]. The coding of qualitative data has been described as a decision-making process through discovering what works under specific circumstances [27] and presenting a relationship between data and the study topic [24]. This procedure should lead to a deeper understanding of the themes, identification of similarities, and interpretation of collective responses.

The results indicated most of the participants reported that changes were driven in the department by senior practitioners (band 6 or above), but a substantial part of the candidates described that anyone has the potential to lead a team, especially when it comes to the initiative and ability to make improvements. These findings were in disagreement with the view which identified the surgeon as the leader in the operating theatre [28], but they matched the ideas of leadership in this environment as being either represented by a mixture of figures [29] and the inclusive option presented by Minehart et al. (2020) [30].

The findings built on previous studies [29,30] and confirmed the main characteristic of a leader was having clear communication, but broadly the adjectives described a leader as an honest and reliable person, who focused on empowering others and supporting the team. However, our participants tended to follow recurrent characteristics for leadership throughout the answers provided to the survey, even if some nurses were not consistently adhering to a single type of leadership (shared, collaborative, ethical, authentic, or servant), but it presented a picture where a mixture of the moral and ethical behaviours coexisted at the same time, reflecting the best nursing leadership.

Furthermore, the answers suggested that even if most candidates felt there were possibilities offered to them by the Trust regarding access to wellbeing services, more help from the management would have been beneficial and appreciated. The overall well-being was impacted by the crisis they were immersed in at work, which in the critical areas meant caring for dying patients, without having the training to provide high standards of care, facing the unknown, and in some cases living with the effects of PTSD. The data showed that there were different ways that improved well-being, such as feeling appreciated, and multiple participants mentioned directly or indirectly teamwork and peer support as key to overcoming difficult times.

The immediate changes included four areas that the staff reported as impacted by the pandemic early in the days.

The constant inflow of different guidelines and policies and the application of patient pathways and working conditions required staff to be able to constantly adapt. The guidelines and policies (such as the COVID-19 Recovery plan 2021 and the Elective Recovery Plan 2022) [31,32], which were published at the national level, have directly influenced the Trust governance and locally impacted on staff management, clinical guidance, and patient care [33].

Another change reported was the implementation of PPE in clinical practice and everyday life. All staff needed to wear PPE, which included an FFP3 mask, a gown, a visor or goggles, and gloves at all times, while caring for sick patients and had to don and doff every time they were entering or exiting the clinical areas. This also was reported to have had an impact on their ability to deal with changes in sensibility (gloves vs. bare hands), their hydration (due to limited toilet breaks available), and body imbalances (skin rashes from gloves worn for long hours, gowns preventing perspiration of the skin, and pressure sores on the face caused by masks or respirators) [7].

Redeployment was seen by many as one of the most difficult parts to overcome, as many staff were moved to a different area, away from theatres, and were asked to just deal with a completely different environment, without having a say (feeling powerless) and without knowing when the end was. The process of redeployment was one of the main failures of leadership in dealing with the acuity of the pandemic and being unprepared to adequately support the staff [34].

Participants reported the overall situation had greatly impacted their well-being, not being able to rest or recharge, and impacting their personal world. This also affected the way of working in the elective orthopaedic theatre department, mainly due to the mental impact and the increased workload and the changes in patients’ pathways and the conditions in which the staff worked. Even if the findings matched the literature in regard to the increased levels of stress, anxiety, and insomnia [35], the answers brought to light the admirable flexibility, adaptability, and resilience demonstrated by the theatre practitioners, not only throughout the pandemic but also when the elective activity restarted.

The theme of delayed changes grouped some issues which were perceived by the staff after the acuity of COVID-19, for the majority when the elective activity restarted. The perceived increase in the workload and the organisational pressure to deliver targets set up at the national level was reported unanimously by the registered and unregistered practitioners. The findings presented indicated a disconnection between what the services required and what the staff felt was needed. The focus of the “Recovery Plan” (NHS England, 2021) [32] is on the need to increase the number of cases to achieve 104% service delivery compared to 2019, to reduce the current waiting lists. The need to increase activity did not specifically address how Trusts can achieve the target while looking after staff’s wellbeing and supporting managers in delivering the service. The staff perspective that emerged from the answers offered ideas on how staff recruitment and retention and the need for investment in robust training programs can not only boost morale but also help the department to be more efficient.

Practitioners reported the impact of work not only on a mental level but also on a physical one; many candidates reported being often tired and some staff who were affected by COVID-19 reported still having long-term symptoms.

The results of the survey were in agreement with the national data (NHS England, 2020) in regard to the gravity of staff shortage [3]. The staff noticed the situation has worsened in the department, due to the increased sickness levels of colleagues and the suspension of recruiting during the acute phase of the pandemic in conjunction with the staff leaving their area and at times the healthcare sector. Staff reported issues around the number of vacancies unfilled, but also the lack of skilled practitioners. This finding was in line with the data presented by the Centre for Perioperative Care (2021) [33] with the demand for knowledgeable staff to prevent cancellations and to effectively troubleshoot their impact on the quality of services offered [36].

All candidates, at different points of the survey, indicated the importance of training and the risks related to the lack of it. Staff also highlighted the benefits of receiving adequate and up-to-date training not only for the treatment of patients or efficiency of the service, but also on a personal level to boost their confidence and to feel valued. Education was one of the key components for organisations to support nurses in their personal and professional development. Specialised training allows practitioners to ensure high level of care in clinical practice, which directly impact patients’ outcomes, peer support (including mentoring and coaching), and maintenance of safety levels.

### 4.1. Implications for Nursing Practice

The recommendations produced suggest actions to be considered by nursing leadership to address the objectives:

(1) Each staff member should have regular meetings with the management so that there can be a documented record of staff wellbeing.
(2) Staff should not only be kept updated on changes impacting their practice, but also be involved in the decision-making when possible.
(3) Managers should be actively involved in development opportunities, including recruitment and retention of staff.

### 4.2. Limitations of the Study

This study acknowledges some limitations. The sample used for the data collection comes from the nurses and healthcare assistants from a single department, which may limit the possibility for the findings to be applicable to other organizations or similar departments in other fields. Additionally, time restrictions meant that the sample size and the questions asked were limited, to allow elaboration. Furthermore, the use of open-ended survey as the methodology for the collection of qualitative data (excluding observations, interviews, and group discussions) was also influenced by the restricted resources available. And finally, the use of subjective data for the survey can be impacted by unconscious bias.

## 5. Conclusions

Based on the thematic analysis of the qualitative data collected in the close-ended survey, it can be concluded that, according to the 20 participants, there are many aspects to consider when nursing management is leading a theatre team. Some important factors for nursing management are the need for constant communication with their staff, building honest relationships, and being a reliable leader, focused on empowering others and supporting the team.

The challenges related to the COVID-19 pandemic, such as the frequent changes in practice and redeployments in unfamiliar departments, had a direct impact on staff wellbeing.

When staff returned to the theatre department, they reported an increased workload and organisational pressure. The shortage of skilled staff and training available felt like added strain on the team.

Overall, the strongest subjects recurring in the findings was the need for nursing leadership to focus on training, to act on staffing shortages, and to be mindful of staff well-being.

## Figures and Tables

**Table 1:** Survey questions.

Subjects	Questions
Exploring leadership	Who do you look to for leadership in your workplace? Who is driving changes in the elective orthopaedic theatre department? If you could select the top 3 characteristics of a leader, what would they be?
About your current experience	4. What is/are the main change(s) in your role since the pandemic? (if no change, please state N/A) 5. Is your work-life balance impacting your daily performance? If yes, please describe how. 6. How is the nursing management supporting you in your role? (if no support, please state N/A)
What the future should look like	7. What steps could be taken in the department to improve the working environment? 8. What factors do you think in the past have contributed to worsened staff satisfaction and increased staff leaving? 9. How would you improve staff satisfaction and retention?

**Table 2:** Coding table.

Themes	Sub-Themes			
Immediate changes	Guidelines and Policies	Personal Protective Equipment	Redeployment	Wellbeing
Delayed changes	Increased Workload	Organizational Pressure	Transfer/Change of unit	Physical health
Pre-existing conditions	Staff shortage	Training	Development and support	Opportunities for promotion

## Data Availability

The data presented in this study are available on request from the corresponding author due to privacy reasons.

## Public Involvement Statement

No public involvement in any aspect of this research.

## Guidelines and Standards Statement

This manuscript was drafted against the COREQ guidelines for qualitative for research (https://www.equator-network.org/reporting-guidelines/coreq/ accessed on 1 January 2024).

## References

[1] WHO COVID-19 Dashboard. https://data.who.int/dashboards/covid19/cases?n=c.

[2] Iacobucci G. (2020). COVID-19: All Non-Urgent Elective Surgery Is Suspended for at Least Three Months in England. BMJ.

[3] Coronavirus » Important and Urgent—Next Steps on NHS Response to COVID-19. https://www.england.nhs.uk/coronavirus/wp-content/uploads/sites/52/2020/03/urgent-next-steps-on-nhs-response-to-covid-19-letter-simon-stevens.pdf.

[4] COVID-19: Impact of the Pandemic on Healthcare Delivery. https://www.bma.org.uk/advice-and-support/covid-19/what-the-bma-is-doing/covid-19-impact-of-the-pandemic-on-healthcare-delivery.

[5] House of Commons Committee Public Accounts. NHS Backlogs and Waiting Times in England: Forty-Fourth. https://committees.parliament.uk/work/1582/nhs-backlogs-and-waiting-times/publications/.

[6] Sultan A.A., Acuña A.J., Samuel L.T., Rabin J.M., Grits D., Gurd D.P., Kuivila T.E., Goodwin R.C. (2020). Utilization of Telemedicine Virtual Visits in Pediatric Spinal Deformity Patients: A Comparison of Feasibility and Patient Satisfaction at a Large Academic Center. J. Pediatr. Orthop..

[7] Al-Jabir A., Kerwan A., Nicola M., Alsafi Z., Khan M., Sohrabi C., O’Neill N., Iosifidis C., Griffin M., Mathew G. (2020). Impact of the Coronavirus (COVID-19) Pandemic on Surgical Practice—Part 2 (Surgical Prioritisation). Int. J. Surg. Lond. Engl..

[8] Biz C., Buffon L., Marin R., Petrova N. (2016). Orthopaedic Nursing Challenges in Poly-Traumatised Patient Management: A Critical Analysis of an Orthopaedic and Trauma Unit. Int. J. Orthop. Trauma Nurs..

[9] Alsadaan N., Salameh B., Reshia F.A.A.E., Alruwaili R.F., Alruwaili M., Awad Ali S.A., Alruwaili A.N., Hefnawy G.R., Alshammari M.S.S., Alrumayh A.G.R. (2023). Impact of Nurse Leaders Behaviors on Nursing Staff Performance: A Systematic Review of Literature. Inq. J. Health Care Organ. Provis. Financ..

[10] Busse R. (2014). Comprehensive Leadership Review-Literature, Theories and Research. Adv. Manag..

[11] Lemoine G.J., Hartnell C.A., Leroy H. (2019). Taking Stock of Moral Approaches to Leadership: An Integrative Review of Ethical, Authentic, and Servant Leadership. Acad. Manag. Ann..

[12] Zhu J., Liao Z., Yam K.C., Johnson R.E. (2018). Shared Leadership: A State-of-the-Art Review and Future Research Agenda. J. Organ. Behav..

[13] Eva N., Robin M., Sendjaya S., van Dierendonck D., Liden R.C. (2019). Servant Leadership: A Systematic Review and Call for Future Research. Leadersh. Q..

[14] Andrade C. (2021). The Inconvenient Truth about Convenience and Purposive Samples. Indian J. Psychol. Med..

[15] Vehovar V., Toepoel V., Steinmetz S. (2016). Non-Probability Sampling. The Sage Handbook of Survey Methodology.

[16] Rampin R., Rampin V. (2021). Taguette: Open-Source Qualitative Data Analysis. J. Open Source Softw..

[17] Braun V., Clarke V. (2022). Toward Good Practice in Thematic Analysis: Avoiding Common Problems and Be(Com)Ing a Knowing Researcher. Int. J. Transgender Health.

[18] Acocella I. (2012). The Focus Groups in Social Research: Advantages and Disadvantages. Qual. Quant..

[19] Errasti-Ibarrondo B., Jordán J.A., Díez-Del-Corral M.P., Arantzamendi M. (2018). Conducting Phenomenological Research: Rationalizing the Methods and Rigour of the Phenomenology of Practice. J. Adv. Nurs..

[20] Terry G., Hayfield N., Clarke V., Braun V. (2017). Thematic Analysis. J. Posit. Psychol..

[21] Braun V., Clarke V. (2006). Using Thematic Analysis in Psychology. Qual. Res. Psychol..

[22] Nowell L.S., Norris J.M., White D.E., Moules N.J. (2017). Thematic Analysis: Striving to Meet the Trustworthiness Criteria. Int. J. Qual. Methods.

[23] Braun V., Clarke V. (2021). One Size Fits All? What Counts as Quality Practice in (Reflexive) Thematic Analysis?. Qual. Res. Psychol..

[24] Gibson W.J., Brown A. (2009). Working with Qualitative Data.

[25] Belotto M. (2018). Data Analysis Methods for Qualitative Research: Managing the Challenges of Coding, Interrater Reliability, and Thematic Analysis. Qual. Rep..

[26] Campbell J.L., Quincy C., Osserman J., Pedersen O.K. (2013). Coding In-Depth Semistructured Interviews: Problems of Unitization and Intercoder Reliability and Agreement. Sociol. Methods Res..

[27] Elliott V. (2018). Thinking about the Coding Process in Qualitative Data Analysis. Qual. Rep..

[28] Arnold D., Fleshman J.W. (2020). Leadership in the Setting of the Operating Room Surgical Team. Clin. Colon Rectal Surg..

[29] Agnoletti V., Gambale G., Meineri M. (2015). Operating Room Leadership: Who Is the One?. J. Anesth. Clin. Res..

[30] Minehart R.D., Foldy E.G., Long J.A., Weller J.M. (2020). Challenging Gender Stereotypes and Advancing Inclusive Leadership in the Operating Theatre. Br. J. Anaesth..

[31] NHS England » NHS Sets out COVID-19 Recovery Plan for Patient Care and Staff Wellbeing. https://www.england.nhs.uk/2021/03/nhs-sets-out-covid-19-recovery-plan-for-patient-care-and-staff-wellbeing/.

[32] Delivery Plan for Tackling the COVID-19 Backlog of Elective Care. https://www.england.nhs.uk/coronavirus/wp-content/uploads/sites/52/2022/02/C1466-delivery-plan-for-tackling-the-covid-19-backlog-of-elective-care.pdf.

[33] Tackling the Elective Surgery Backlog. https://www.cpoc.org.uk/sites/cpoc/files/documents/2021-09/CPOC-Perioperative-Care-Solutions-FINAL.pdf.

[34] Kennedy E., Kennedy P., Hernandez J., Shakoor K., Munyan K. (2022). Understanding Redeployment During the COVID-19 Pandemic: A Qualitative Analysis of Nurse Reported Experiences. SAGE Open Nurs..

[35] Rossi R., Socci V., Pacitti F., Di Lorenzo G., Di Marco A., Siracusano A., Rossi A. (2020). Mental Health Outcomes Among Frontline and Second-Line Health Care Workers During the Coronavirus Disease 2019 (COVID-19) Pandemic in Italy. JAMA Netw. Open.

[36] Farr M., Cressey P. (2015). Understanding Staff Perspectives of Quality in Practice in Healthcare. BMC Health Serv. Res..

